# Laparoscopic Heller-Dor is an effective long-term treatment for end-stage achalasia

**DOI:** 10.1007/s00464-022-09696-8

**Published:** 2022-10-10

**Authors:** Renato Salvador, Giulia Nezi, Francesca Forattini, Federica Riccio, Arianna Vittori, Luca Provenzano, Giovanni Capovilla, Loredana Nicoletti, Lucia Moletta, Elisa Sefora Pierobon, Michele Valmasoni, Stefano Merigliano, Mario Costantini

**Affiliations:** Department of Surgical, Oncological and Gastroenterological Sciences, University of Padova, Padova, Italy

**Keywords:** Achalasia, End-stage achalasia, Sigmoid esophagus, Laparoscopic Heller-Dor

## Abstract

**Background:**

The end-stage achalasia is a difficult condition to treat, for the esophageal diameter and conformation of the gullet, that may progress to a sigmoid shape. The aim of this study was to examine the outcome of Laparoscopic Heller-Dor in patients with end-stage achalasia, comparing them with patients who had mega-esophagus without a sigmoid shape.

**Methods:**

From 1992 to 2020, patients with a diagnosis of sigmoid esophagus, or radiological stage IV achalasia (the SE group), and patients with a straight esophagus larger than 6 cm in diameter, or radiological stage III achalasia (the NSE group), were all treated with LHD. The two groups were compared in terms of patients’ symptoms, based on the Eckardt score, and on barium swallow, endoscopy and manometry performed before and after the treatment. The failure of the treatment was defined as an Eckardt score > 3, or the need for further treatment.

**Results:**

The study involved 164 patients: 73 in the SE group and 91 in the NSE group. No intra- or postoperative mortality was recorded. The median follow-up was 51 months (IQR 25–107). The outcome was satisfactory in 71.2% of patients in the SE group, and in 89% of those in the NSE group (*p* = 0.005).

**Conclusions:**

SE is certainly the worst condition of the disease and the final outcome of LHD, in term of symptom control, is inferior compared to NSE. Despite this, almost 3/4 of the SE patients experienced a significant relieve in symptoms after LHD, which may therefore still be the first surgical option to offer to these patients, before considering esophagectomy.

Achalasia is a rare disease affecting esophageal motility [1]. According to the latest Chicago Classification of motility disorders, which considers high-resolution manometric findings, achalasia is characterized by a high median integrated relaxation pressure (IRP) combined with failed peristalsis or spasm [2]. Its treatment aims for symptom relief and may be based on pharmacological therapies (calcium blockers or nitrates), endoscopic treatments [pneumatic dilation (PD) or Botox injections] or surgery (using the Heller-Dor technique or POEM) [3], depending on the patient’s characteristics and the stage of the disease. The natural history of achalasia features a possible continuous evolution of the manometric patterns involved [3], along with changes in the shape of the esophagus, which becomes gradually enlarged, ultimately acquiring a sigmoid shape.

While laparoscopic myotomy has changed the overall approach to this rare disease [4, 5] since the 1990s, the end-stage achalasia is a condition hard to treat because of the esophageal diameter and its conformation (sigmoid shape). Historically, this condition was often treated with esophagectomy. Only a few studies in the literature evaluated the outcome of Laparoscopic Heller-Dor (LHD) in end-stage achalasia patients, reporting good control of symptoms in 70–90% of patients [6–8].

The aim of our study was to analyze our single-center experience to ascertain the role of LHD for long-term symptom relief in patients with radiological stage IV achalasia, as compared with the results obtained in patients with a dilated, but not sigmoid esophagus.

## Materials and methods

Between 1992 and 2020, a total of 1323 patients were treated with LHD for esophageal achalasia at the Department of Surgical, Oncological, and Gastroenterological Sciences at the University of Padova (Italy). The 164 patients involved in the present study included: 73 patients diagnosed with end-stage achalasia (radiological stage IV achalasia) with a sigmoid esophagus, who formed the SE group; and 91 patients with a straight esophagus larger than 6 cm in diameter (radiological stage III achalasia), who formed the NSE group. Patients with a history of surgical or endoscopic myotomy were excluded from the study.

The study was approved by the Research Committee of the Department of Surgical, Oncological, and Gastroenterological Sciences—University of Padova (Protocol: DOR2023591). It was a retrospective study and therefore consent form was not necessary.

### Preoperative assessment

The patients’ demographic and clinical data were collected prospectively in a dedicated database, together with details of previous treatments, manometric findings, and symptom scores before surgery. Before the treatment, all patients underwent:

- Endoscopy to rule out malignancies and/or other esophageal diseases;
- Barium swallow to examine the diameter and shape of the esophagus;
- Esophageal manometry (with the conventional or high-resolution technique) to assess the pattern and manometric parameters.

Symptoms were quantified using the Eckardt score [9], as shown in Table 1.

**Table 1:** Eckardt symptom score

Score	Chest pain	Regurgitation	Dysphagia	Weight loss (kg)
0	Never	Never	Never	0
1	Occasional	Occasional	Occasional	< 5
2	Daily	Daily	Daily	5–10
3	Each meal	Each meal	Each meal	> 10

### Surgical technique

**Fig. 1:**
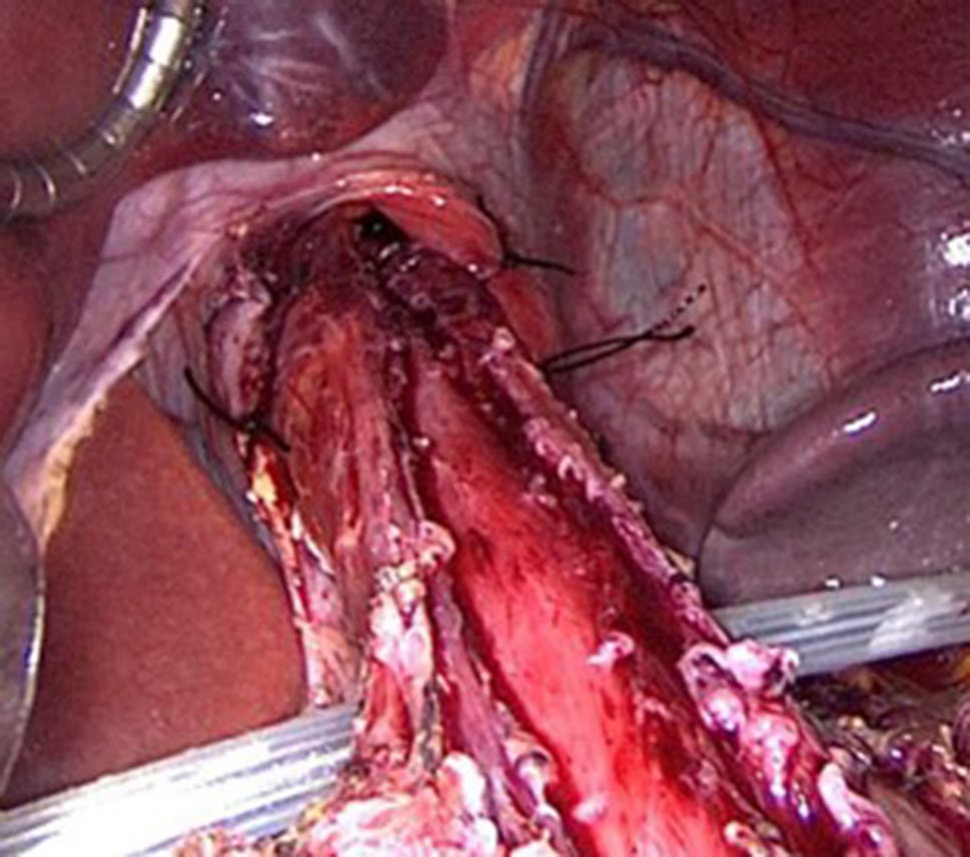
The pull-down technique: the esophagus is isolated from the pillars and the dissection is extended about 10 cm upwards. The surgeon applies two or more stitches on each side of the esophageal wall to anchor it to the pillars

The surgical technique for Heller-Dor myotomy has been described in detail elsewhere [10]. All the surgeons involved completed the procedure in the same fashion for all patients. A pull-down technique was added for some patients with sigmoid esophagus to straighten the esophageal axis. After circling the gastro-esophageal junction using a string (Easy-Flow/Penrose drain), a length of approximately 10 cm of the lower mediastinal esophagus was isolated. Two or more stitches were applied on each side, then tied to anchor the wall of the esophagus to the diaphragmatic pillars [11]. After verticalizing the esophageal axis, the Heller-Dor myotomy was performed as already reported (Fig. 1).

### Postoperative assessment

Postoperative data reflect the center’s protocol on follow-up, which is based on barium swallow after 1-month, esophageal manometry and 24-h pH monitoring after 6 months, and EGDS after 12 months, then every 2 years [12]. Symptoms were scored every time patients undergo a clinical examination. During the drafting of the present study, patients were also contacted telephonically to obtain an update on their clinical conditions. Treatment failure was defined as the persistence or recurrence of an Eckardt score > 3, or the need for further treatment (pneumatic dilation, POEM, redo-myotomy or esophagectomy) [13].

### Statistical analysis

Continuous data were expressed as medians and interquartile ranges (IQR), and categorical data as numbers and percentages. Comparisons were performed using the Mann–Whitney test, the Chi-square test, and Fisher’s exact test. A *p* < 0.05 was considered statistically significant.

## Results

All patients in the SE and NSE groups underwent LHD for esophageal achalasia during the study period, with a median follow-up of 51 months (IQR 25–107); 3 patients were lost to follow-up, and 8 died (5 in the SE group, and 3 in the NSE group). One SE patient developed a squamous cell esophageal carcinoma. After missing several follow-up endoscopies, this patient presented with advanced cancer 8 years after LHD and died 14 months later, despite neo-adjuvant therapy and esophageal resection.

Sex, age and preoperative symptom scores were similar in the two groups. There was also no statistically significant difference between the numbers of patients with a history of endoscopic treatments (PD or Botox injections, or both): 23/73 (32%) in the SE group and 22/91 (24%) in the NSE group (*p* = 0.41). The two groups differed, however, in terms of symptom duration: 60 months (IQR 24–144) for the SE group and 25 months (IQR 12–89) for the NSE group (*p* = 0.002). Manometric findings also differed between the two groups: IRP and LES basal pressure were higher in the NSE group (29 mmHg, IQR 23–39 and 33 mmHg, IQR 25–47, respectively) than in the SE group (15 mmHg, IQR 3–29 and 27 mmHg, IQR 15–34, respectively) (*p* < 0.01). The patients’ preoperative and demographic data are summarized in Table 2.

**Table 2:** Preoperative demographic, clinical and manometric data

	SE	NSE	*p* value
Patients (*n*)	73	91	–
Age (years)*	53 (39–59)	52 (42–66)	0.17
Sex (M:F)	38:35	55:36	0.36
Symptom duration (months)*	60 (24–144)	25 (12–89)	**0.002**
Preoperative symptom score (Eckardt score)*	7 (5–8)	7 (3–9)	0.27
Previous endoscopic treatment (%)	23 (32%)	22 (24%)	0.38
IRP (mmHg)*	15 (3–29)	29 (23–39)	**0.01**
LES basal pressure (mmHg)*	27 (15–34)	33 (25–47)	**0.002**

The bold highlights *p* < 0.05

*IRP* integrated relaxation pressure, *LES* lower esophageal sphincter

*Data are shown as median (IQR)

**Fig. 2:**
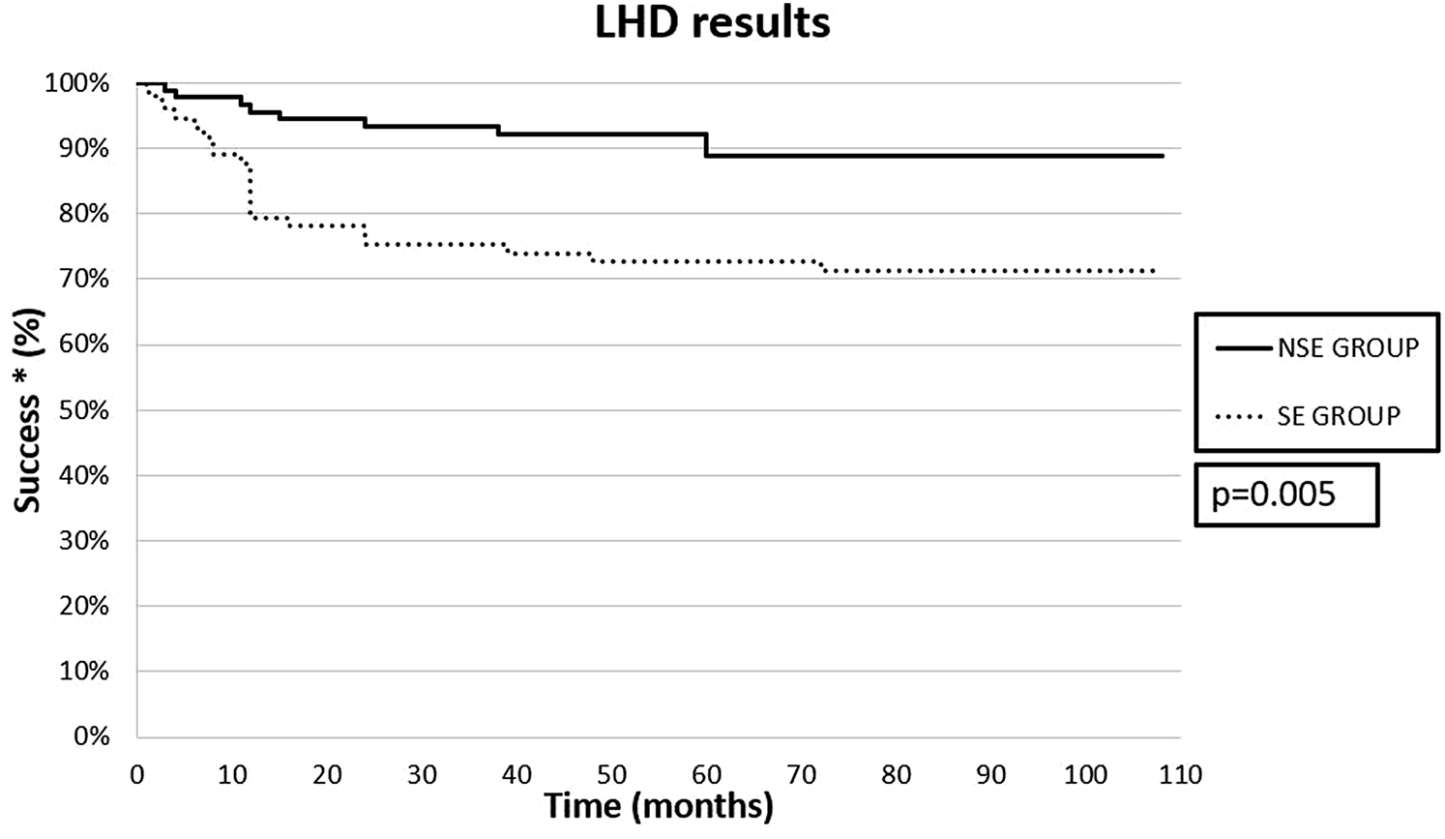
Kaplan–Meier curves for a positive outcome in the two groups: the probability of end-stage achalasia patients having a persistently good outcome after LHD was higher than 71% at 9 years after surgery. *Success was defined as Eckard score < 3 and no need for further treatment

The surgical procedure was completed laparoscopically in all patients. No intra- or postoperative mortality was recorded. Perioperatively, there were 7 intraoperative esophageal perforations (4.3%), 2 in the SE group and 5 in the NSE group (*p* = 0.46). All lesions were detected and repaired intra-operatively and without further consequences. No other intra- or postoperative complications (such as hemorrhage, pneumothorax, pneumonia, or pleural effusion) were recorded. Symptom scores after LHD were similar in the two groups: 1 (IQR 0–2) in the SE group, and 1 (IQR 0–2) in the NSE group (*p* = 0.20). The outcome of the surgical treatment was considered satisfactory (Eckard score < 3 and or no further treatments) for 52 patients in the SE group (71.2%), and 81 patients in the NSE group (89%) (*p* = 0.005). (Fig. 2).

**Fig. 3:**
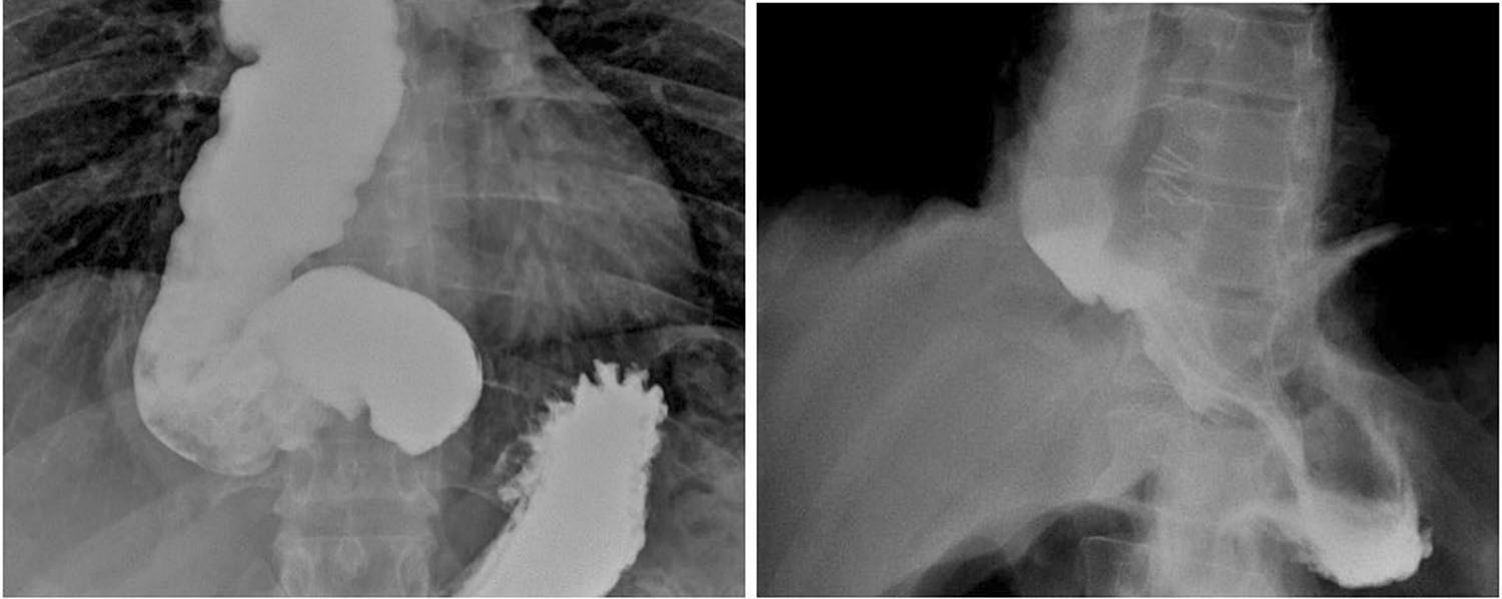
Pre- and post-operative barium swallows using the pull-down technique

The pull-down technique was used in 18 patients (24.6%) in the SE group. All patients experienced a reduction in their symptom scores after surgery, but the failure rate was 30.9% (17/55) after classic LHD, and 22.2% (4/18) after LHD with the pull-down technique (*p* = 0.56). (Fig. 3). The postoperative results are shown in Table 3.

**Table 3:** Postoperative manometric and clinical data

	SE	NSE	*p* value
Postoperative symptom score (Eckardt score)*	1 (0–2)	1 (0–2)	0.20
IRP (mmHg)*	7 (4–9)	10 (7–13)	**0.01**
LES basal pressure (mmHg)*	12 (9–20)	14 (9–20)	0.35
Satisfactory outcome (%)^#^	52 (71.2%)	81 (89%)	**0.005**
Time to failure (months)*	11 (6–13)	20 (11–54)	0.10
Abnormal findings on 24-h pH-monitoring (%)	14%	8.7%	0.49

The bold highlights *p* < 0.05

*LES* lower esophageal sphincter, *IRP* integrated relaxation pressure

*Data are shown as median (IQR)

^#^Satisfactory outcome was defined as Eckard score < 3 and no need for further treatment

All failures occurring in the NSE group (10 patients) were treated with endoscopic PD, with an overall success rate of 98.9%. As concerns the failures in the SE group (21 patients), 5 patients refused additional treatment, while all the others underwent endoscopic PD as the first retreatment, with a success of 56% (9/16). No dilation-related complications were recorded. The overall success rate of a combination of LHD and PD in the SE group was 83.5%. Further treatments for persistent failures included: a redo-myotomy in 4 patients; a Botox injection in one; POEM in one; and esophagectomy in one.

There was no statistically significant difference in the median time to failure between the two groups: 20 months (IQR 11–54) among the NSE patients and 11 months (IQR 6–13) among the SE patients (*p* = 0.10). Considering the patients whose LHD procedure failed, the success rate in the SE group was lower among patients who had a history of endoscopic treatments (12/23; 52%) than in those who did not (40/50; 80%) (*p* = 0.02). In the NSE group, on the other hand, the success rate was much the same for patients who had previously undergone endoscopic treatments (19/22; 86%) and for those who had not (62/69; 90%) (*p* = 0.70).

Postoperative 24-h pH monitoring produced similar results in both groups: an abnormal acid exposure was recorded in 14% of patients in the SE group, and in 8.7% of patients in the NSE group (*p* = 0.49).

## Discussion

Since the etiology of esophageal achalasia is still unknown, all the therapies currently used to relieve patients’ symptoms aim to provide palliative care and the treatment strategy needs to be tailored to each patient’s characteristics [1, 3]. About 5% of cases of achalasia evolves towards a sigmoid mega-esophagus, that constitutes the end-stage of the disease [14, 15]. This condition can be encountered in first-diagnosed patients or even in treated patient, despite any previous treatments, even if effective in relieving symptoms, but unable to restore the function of the esophagus and esophageal sphincter to normal [14, 16]. The end-stage disease is certainly a condition that does not have a single point of view and it is really difficult to treat, because of the esophageal diameter and conformation (sigmoid shape). Evidence-based data regarding the finest surgical options for these patients are lacking, and treatment follows the experience and surgeon’s preference [3]. Moreover, the 2018 ISDE guidelines do not recommend a specific therapeutic approach to end-stage achalasia, thus confirming the difficulty to treat this cohort of patients [17, 18].

The most important finding of our study is that long-term satisfactory outcome can be achieved by LHD in a good proportion of patients with dilated, decompensated esophagus, even in its final stage of elongation and angulation, with the ultimate sigmoid-shaped appearance. Only a few studies in the literature (with several limitations) examined the outcome of laparoscopic Heller myotomy in end-stage achalasia patients. In 1999, Patti et al. [19] reported on their experience with LHD in different stages of achalasia, claiming good results in all the 7 patients with sigmoid-shaped esophagus they treated, at a median follow-up of 25 months. After this somewhat enthusiastic paper, Sweet et al. [6] reported on the treatment of 20 end-stage achalasia patients with LHD: they found a significant improvement in the symptoms of 91% of patients with a sigmoid esophagus. The main limitation of this study concerns the short follow-up, which was less than 2 years for some patients. Mineo et al. [8] also assessed the clinical outcomes of surgical myotomy in 14 patients with sigmoid mega-esophagus. They reported a positive outcome in 71% patients with a median follow-up of 85 months. In all these studies, the operation was reportedly no more technically demanding than in patients with earlier-stage achalasia, the complication rates were much the same, and the relief of dysphagia was just as good. Our study confirmed their results in this cumbersome condition of the disease in terms of success of treatment and symptoms relief. The outcome of laparoscopic surgical myotomy in a large number of patients and with a long follow-up, as palliative treatment, was acceptable in more than 70% of our end-stage achalasia patients. And this without major complications or mortality and a intraoperative perforation rate during myotomy not different to that historically recorded in earlier-stages of the disease (less than 3%) [20].

Youn et al. [21] recently assessed the outcome of POEM for end-stage achalasia, reviewing the technical details and clinical outcomes of this procedure in patients with sigmoid esophagus. It emerged that submucosal fibrosis (a consequence of food stasis) could influence the correct performance of the submucosal tunnel and the maintenance of its appropriate alignment. The Authors found that placing the submucosal tunnel as deeply as possible could help to maintain its correct aboral direction by ensuring that it was perpendicular to the circular muscle fibers. Hu et al. [22] reported that POEM was feasible and effective in the short term (96.8%) also in patients with a sigmoid esophagus. However, they observed rates of mucosal injuries (37.5%) and intra- or postoperative complications higher than in patients with radiological stage III achalasia. They consequently concluded that these patients with advanced-stage disease are particularly challenging, and any POEM procedure should be performed only by highly experienced operators. More studies with long-term follow up are however necessary before clearly defining the role of POEM in end-stage achalasia.

Esophagectomy may be recommended as a first approach to treat end-stage achalasia by some authors who believe that the chances of a positive final outcome of surgical myotomy are much reduced when the esophagus has a sigmoid shape, reflecting a permanent damage of the gullet [23, 24]. Many others, as we do, judge that a conservative approach should always be offered first, however, given the good outcome observed in a large percentage of cases. A native esophagus, even if impaired in function, is always better than any substitutes, being a tubulised stomach or a colonic segment. Esophagectomy is a major surgical undertaking associated with significant morbidity and even mortality [14, 23]. The most common complications, which affect approximately 30% of patients, include anastomotic leakage, laryngeal nerve injury, wound infection, bleeding, chylothorax, tracheal tear, and pneumonia [14, 25]. There may also be recurrent dysphagia, due to stenosis of the anastomosis, in 50% of cases. The mortality rate for esophagectomy to treat sigmoid esophagus is reportedly around 2%, even when the procedure is performed by an experienced surgeon [23].

**Fig. 4:**
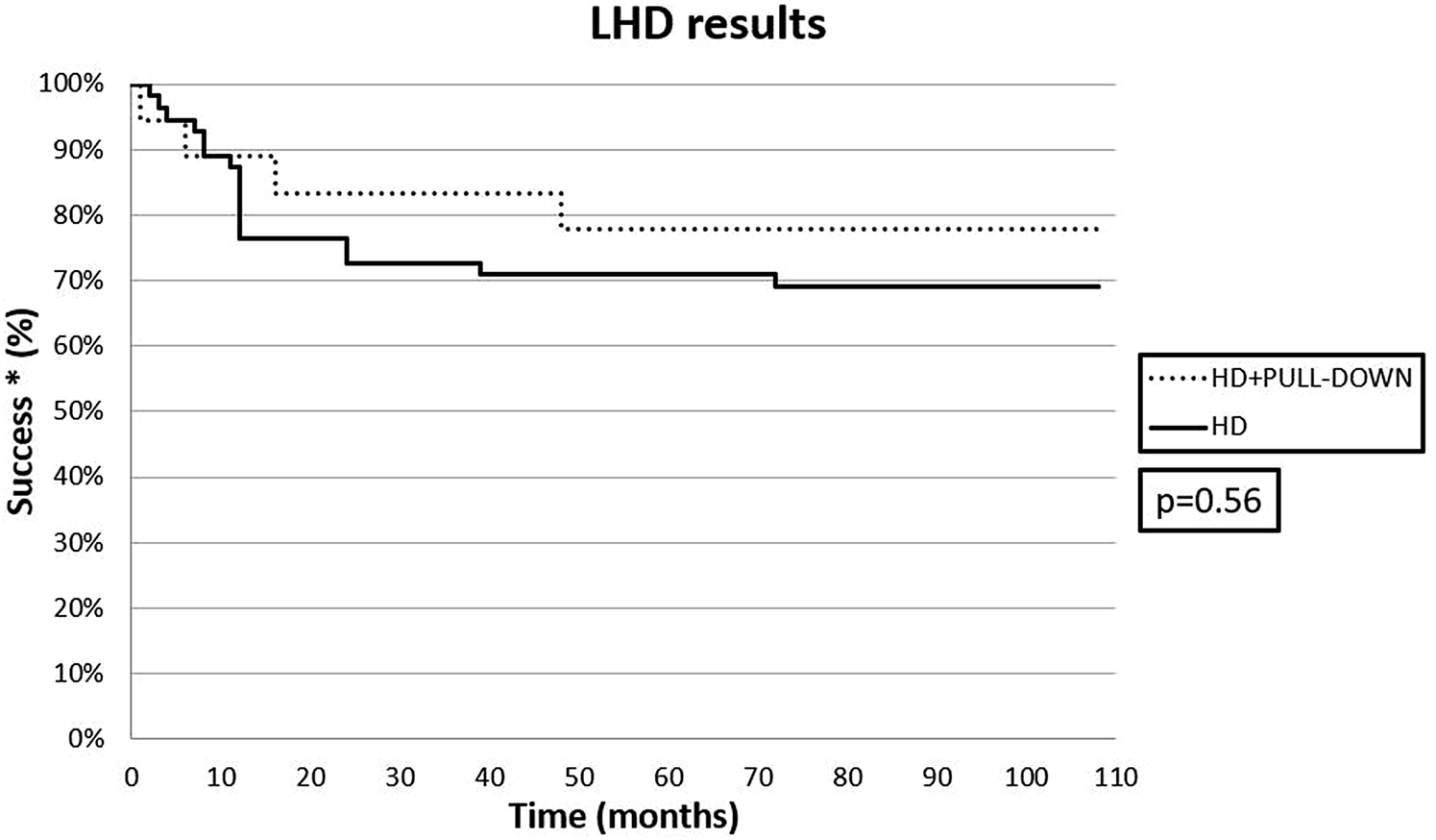
Kaplan–Meier curves for a positive outcome in end-stage achalasia patients after classic LHD and LHD with the pull-down technique. *Success was defined as Eckard score < 3 and no need for further treatment

Our study also compared the results obtained with LHD in cases of dilated but straight esophagus as opposed to sigmoid esophagus (i.e.: radiological stage III vs stage IV achalasia). As expected, patients with the former had a better outcome than patients with the latter, comparable to that usually obtained in earlier stages of achalasia. However, by considering the proportion of stage IV patients that underwent an esophageal straightening procedure (18/73, 24.7%), the outcome results were much better, approaching those obtained in stage III patients. This confirms the findings of Faccani et al. [11], who used the same pull-down technique on some of their patients. In fact, we demonstrated a greater efficacy of the LHD with pull-down technique with a positive outcome in 77.8% of cases, as compared with 69% of patients treated with traditional LHD. This finding just failed to reach statistical relevance probably for the small number of cases (Fig. 4). Further studies on larger cohorts of end-stage achalasia patients are now needed to confirm the usefulness of the pull-down technique. This finding, however, prompted us to change our surgical strategy in these patients, by adding a straightening procedure to every patient with sigmoid esophagus undergoing LHD, instead of reserving it to selected cases only, with more pronounced angulation of the esophageal longitudinal axis.

Our study also showed a lower long-term palliative efficacy of LHD in end-stage patients when previous traditional endoscopic treatments failed. It may be speculated if the effects of previous endoscopic treatments, causing some fibrotic alterations in the esophageal wall, may jeopardize the efficacy of rescue myotomy, or if previous treatments helped in selecting patients highly refractory to any treatments. Unfortunately, there are no studies in Literature addressing this question. Finally, it may be argued that a dilated, decompensated esophagus, especially if sigmoid in shape, may increase the risk of pathological post-operative reflux for the stasis of any material eventually refluxed from the stomach. It is worth mentioning, however, that 24-h pH-monitoring revealed similar rates of abnormal acid exposure in the distal esophagus after surgery both in SE and NSE groups. And these rates were not dissimilar to the rate of reflux historically reported by our group in a large series of patients [7].

This study has some intrinsic limitations, that need to be mentioned. The main limitation is that it is a retrospective, non-randomized trial. A second limitation concerns the straightening procedure of the esophageal axis performed only in some selected cases. A third limitation may be that different surgeons performed the operations over a long period of time. However, these five surgeons were formed within the same group, the younger surgeons taking full advantage from the experience of the senior ones, and kept performing the operation in the same way, with only minor modifications [7].

## Conclusions

This study confirmed that SE is certainly the worst condition of esophageal achalasia and the final outcome of LHD is inferior when compared to NSE or earlier stages of the disease. Despite this, almost 3/4 of the SE patients experienced a significant relieve in symptoms after LHD. It should therefore be the first surgical option to be offered to patients in such advanced stage of the disease, reserving esophagectomy to failures of such a “conservative” surgical procedure.
